# The mediating role of self-advocacy in family cohesion and adaptability and health promotion in breast cancer survivors

**DOI:** 10.3389/fmed.2025.1556701

**Published:** 2025-03-26

**Authors:** Li Qian, Yan Li, Dan Yue

**Affiliations:** ^1^School of Nursing, North Sichuan Medical College, Nanchong, China; ^2^Department of Education, Chongqing University Three Gorges Hospital, Chongqing, China

**Keywords:** breast cancer, health promotion, self-advocacy, family cohesion and adaptability, mediating effect

## Abstract

**Background:**

Breast cancer is the most common cancer among women. Health-promoting behaviors can enhance the quality of life for breast cancer survivors; however, further research is needed to identify the factors influencing these behaviors.

**Methods:**

A survey was conducted on 238 breast cancer survivors from a tertiary hospital in Chongqing using a general information questionnaire, the Family Cohesion and Adaptability Scale, the Self-Advocacy Scale, and the Health-Promoting Lifestyle Profile II. Descriptive analysis was performed using SPSS 26.0, and the mediating effect of variables was analyzed using the structural equation model in SPSS AMOS 24.0.

**Results:**

The model results revealed positive correlations between family cohesion and adaptability and self-advocacy (*β* = 0.55; *p* < 0.05), between family cohesion and adaptability and health promotion (*β* = 0.42; *p* < 0.05), and between self-advocacy and health promotion (β = 0.43; *p* < 0.05) among breast cancer survivors. Furthermore, self-advocacy partially mediated the relationship between family cohesion and adaptability and health-promoting lifestyles (β = 0.237; *p* < 0.05).

**Conclusion:**

Self-advocacy is a mediator variable in the relationship between family cohesion and adaptability, and the health-promoting lifestyle of breast cancer survivors.

## 1 Introduction

According to the 2020 Global Cancer Report, breast cancer emerged as the most frequently diagnosed cancer among women worldwide, with a global incidence of 2.3 million cases and over 685,000 deaths in 2020, surpassing lung cancer in prevalence (1). Accumulating evidence suggests that unhealthy lifestyle behaviors constitute significant risk factors for cancer development (2). Moreover, such lifestyles exert a detrimental influence on the prognosis of cancer survivors. Specifically, survivors with unhealthy lifestyles exhibit nearly twice the risk of all-cause mortality compared to those adhering to healthier practices (3). The Health-Promoting Lifestyle Profile (HPLP), defined as a multidimensional behavioral pattern aimed at enhancing health, encompasses a series of actions individuals undertake to maintain or improve their health, achieve self-satisfaction, and attain self-actualization (4). Research has demonstrated that health-promoting behaviors among breast cancer survivors not only alleviate symptom clusters resulting from the disease and its treatment but also enhance treatment adherence and overall quality of life in cancer patients (5, 6).

Although a healthy lifestyle has been proven beneficial for cancer survivors, the adherence rate of breast cancer survivors to recommended lifestyle guidelines remains low (7). Hyland et al. (8) found that only 7.6% of survivors met all six criteria of healthy behaviors outlined in the lifestyle guidelines for breast cancer survivors. Therefore, it is necessary to further investigate the factors influencing the participation and adherence of breast cancer survivors in health promotion activities.

Self-advocacy refers to the ability of survivors to exercise autonomy in coping with their illness and to prioritize meeting their own needs and desires (9). As a positive psychological factor, self-advocacy is an ongoing process of internalizing skills and resources to support oneself, fulfill needs, and achieve goals. Research has shown that self-advocacy helps survivors adopt proactive coping strategies, thereby motivating health-promoting behaviors (10).

Family cohesion refers to the emotional bonds among family members, while family adaptability refers to the ability of the family system to alter its power structure, role relationships, and relational rules in response to stress (11). A growing body of research indicates that family cohesion and adaptability have long-term positive effects on the maintenance of healthy behaviors, with individuals experiencing higher levels of family cohesion typically exhibiting healthier behaviors (12). However, the interrelationships among health-promoting behaviors, self-advocacy, family cohesion, and adaptability in breast cancer patients remain unclear. Therefore, this study constructed a structural equation model (SEM) to explore the relationships among these factors, aiming to provide support for the future health promotion management of breast cancer survivors.

### 1.1 Conceptual framework and hypotheses

Self-Determination Theory (SDT), a prominent theory within positive psychology, posits that proactive individuals, when their fundamental psychological needs are satisfied, are empowered to evaluate environmental factors and make informed behavioral choices, thereby fostering personal development. This theory has been widely applied in various contexts to facilitate behavioral change among patients (13). In accordance with SDT, external regulation is predictive of short-term behavioral changes, whereas intrinsic motivation is a more robust predictor of sustained, long-term adherence. Consequently, health behavior interventions should prioritize the enhancement of intrinsic motivation to foster engagement and persistence, while also considering the crucial role of the social environment in cultivating optimal motivation (14). Building on these principles, we hypothesize that self-advocacy exerts an influence on health-promoting behaviors and mediates the relationship between family cohesion, adaptability, and these behaviors. The research hypotheses are as follows:

Hypothesis 1: Family cohesion and adaptability are positively correlated with health-promoting behaviors.

Hypothesis 2: Self-advocacy is positively correlated with health-promoting behaviors.

Hypothesis 3: Self-advocacy mediates the relationship between family cohesion and adaptability and health-promoting behaviors.

## 2 Method

### 2.1 Participants

The sample size was determined using a formula appropriate for cross-sectional studies $n=\left(\mu_{\alpha /2}^{2}\sigma^{2}\right)\delta^{2}\text{.}$ Based on pre-survey data, the standard deviation for health-promoting behaviors was 15. Considering practical constraints in sample collection, the allowable error was set at 2, which falls within 0.25 to 0.5 times the standard deviation (15). Accounting for an anticipated 10% invalid response rate, the final calculated sample size was 238 participants. Inclusion criteria were: female gender, age ≥ 18 years, pathological confirmation of breast cancer, and informed consent to participate. Exclusion criteria comprised: terminal illness with impaired self-care abilities, severe chronic conditions impacting daily functioning, and the presence of mental or cognitive disorders, hearing or visual impairments, or any condition hindering cooperation with the survey. Ethical approval for this study was obtained from the hospital ethics committee (approval number: MR-50-24-009092).

### 2.2 Measures

#### 2.2.1 Demographic characteristics questionnaire

The questionnaire included general demographic information (age, gender, marital status, education level, etc.) and disease-related information (disease stage, treatment methods, etc.).

#### 2.2.2 Family intimacy and adaptability scale (Chinese version)

This scale is a translated and revised version of Olson et al.’s (16) Family Intimacy and Adaptability Scale (2nd edition), as modified by Lipeng et al. (17). The instrument requires participants to respond twice, first reflecting their current perceptions of their family dynamics and then describing their ideal family situation. The scale comprises two dimensions: family intimacy (16 items) and family adaptability (14 items), totaling 30 items. Responses are recorded using a 5-point Likert scale, ranging from 1 (“never”) to 5 (“always”). Higher scores indicate greater levels of perceived family intimacy and adaptability. For the purposes of this study, only the participants’ perceptions of their actual family dynamics were examined; therefore, only responses pertaining to their current situations were analyzed. The Cronbach’s *α* coefficient for this scale in the present study was 0.957.

#### 2.2.3 Self-advocacy scale for female cancer patients (Chinese version)

This scale, developed by Hagan et al. (18) and translated into Chinese by Feng et al. (19), was employed to evaluate the self-advocacy levels of Chinese female cancer patients. The Chinese version of the scale encompasses three dimensions: informed decision-making (6 items), effective communication (6 items), and accessing effective social support (6 items), resulting in a total of 18 items. A 6-point Likert scale is used for responses, ranging from “strongly disagree” (1 point) to “strongly agree” (6 points), with higher scores reflecting greater self-advocacy. In the current study, the scale demonstrated a Cronbach’s *α* coefficient of 0.803.

#### 2.2.4 Health-promoting lifestyle profile II

This scale was revised by Walker et al. (20) based on the Health-Promoting Lifestyle Profile (HPLP). The instrument comprises 52 items, distributed across six dimensions: Health Responsibility (9 items), Nutrition (9 items), Physical Activity (8 items), Spiritual Growth (9 items), Stress Management (8 items), and Interpersonal Relationships (9 items). A 4-point Likert scale is employed, with responses ranging from 1 (never) to 4 (always). Consequently, total scores range from 52 to 208, which are categorized into four levels: excellent (172–208), good (132–171), fair (92–131), and poor (52–91). Higher scores are indicative of more positive health behaviors. In the present study, the scale exhibited a Cronbach’s *α* value of 0.873.

### 2.3 Data collection

Data collection for this study was conducted via face-to-face questionnaire surveys between January to June 2024. The participants were breast cancer survivors from a tertiary hospital in Chongqing. Before data collection began, the research team received training to ensure consistent and standardized procedures for distributing and administering the questionnaires. Potential participants were rigorously screened according to pre-defined inclusion and exclusion criteria. Questionnaires were distributed to survivors who met these criteria and provided informed consent, continuing until the target sample size was reached. To accommodate the length of the questionnaire, researchers aimed to conduct surveys when patients were in good physical condition and mentally relaxed. Participants could complete the questionnaire in multiple sessions or withdraw at any time, according to their needs. For participants unable to complete the questionnaire independently, researchers conducted individual interviews, recording responses accurately and avoiding any leading questions. A total of 240 questionnaires were distributed, with 2 deemed invalid, resulting in an effective response rate of 99%. Questionnaires were considered invalid if they met either of these criteria: ① completion time was less than 5 min; ② there were obvious response patterns.

### 2.4 Statistical analysis

SPSS 26 and AMOS 24 were used for data analysis. General information was presented as frequencies and percentages. The total scores and dimension scores of family intimacy and adaptability, self-advocacy, and health-promoting behaviors were expressed as means ± standard deviations. Pearson correlation analysis was used to examine the relationships between variables. Independent sample t-tests and one-way ANOVA were employed to explore the factors influencing health-promoting behaviors. The structural equation model was constructed using AMOS 24.0 and path analysis was conducted to verify the mediating effects using the Bootstrap method. A significance level of *p* < 0.05 was considered statistically significant.

## 3 Results

### 3.1 Demographic characteristics

Among the 238 samples, the majority (62.2%) were between 41–60 years old. Significant differences in health-promoting scores were found among breast cancer survivors with different residential statuses, educational levels, average monthly household incomes, pathological stages, treatment plans, surgical methods, and disease duration (all *p* < 0.05), as shown in Table 1.

**Table 1:** Health promotion scores of breast cancer survivors with different characteristics (*N* = 238).

Variable	*n* (%)	Health promotion (x ± S)	t/F	*P*
Age (years)				
≥61	30 (12.6)	122.37 ± 20.62	0.405	0.668
41–60	148 (62.2)	122 ± 18.39		
≤40	60 (25.2)	124.65 ± 21.07		
Residence				
Rural	121 (50.8)	121.99 ± 18.82	0.35	0.705
Town/County	77 (32.4)	124.23 ± 18.84		
city	40 (16.8)	121.98 ± 21.95		
Living situation				
Living alone	21 (8.8)	114.1 ± 13.22	−2.156	0.032
Not living alone	217 (91.2)	123.55 ± 19.64		
Marital status				
Single	27 (11.3)	126.15 ± 25.59	0.762	0.452
Married	211 (88.7)	122.27 ± 18.41		
Number of children				
0	18 (7.6)	126.22 ± 25.77	0.330	0.719
1	106 (44.5)	122.23 ± 19.48		
≥2	114 (47.9)	122.61 ± 18.13		
Education level				
Elementary or below	105 (44.2)	111.99 ± 14.09	40.616	<0.001
Junior high school	52 (21.8)	123 ± 14.83		
High school	39 (16.4)	130.1 ± 15.14		
College, bachelor’s degree, or above	42 (17.6)	142.31 ± 20.75		
Employment status				
Unemployed	159 (66.8)	123.03 ± 19.61	1.639	0.181
Retired	18 (7.6)	113.39 ± 10.14		
Sick leave	10 (4.2)	125.7 ± 21.31		
Employed	51 (21.4)	124.45 ± 20.11		
Average monthly household income (yuan)				
<3,000	141 (59.2)	121.58 ± 18.71	4.013	0.019
3,000–5,000	66 (27.7)	120.89 ± 17.75		
>5,000	31 (13.1)	131.74 ± 23.21		
Type of insurance				
Self-financed or others	11 (4.6)	119.36 ± 15.14	0.325	0.723
Residents’ medical insurance	149 (62.6)	122.36 ± 19.31		
Employee medical insurance	78 (32.8)	123.86 ± 20.03		
Breast cancer staging				
Stage I	82 (34.5)	134.7 ± 17.76	31.185	<0.001
Stage II	72 (30.3)	123.65 ± 16.55		
Stage III	51 (21.4)	111.84 ± 11.28		
Stage IV	33 (13.8)	107.7 ± 19.29		
Treatment methods				
Surgery alone	116 (48.7)	125.91 ± 19.44	3.753	0.012
Chemotherapy or radiotherapy	34 (14.3)	118.15 ± 16.95		
Endocrine therapy	9 (3.8)	133.11 ± 15.72		
Combination therapy	79 (33.2)	118.81 ± 19.47		
Surgical approach				
Breast conservation surgery	79 (33.2)	127.28 ± 17.17	3.724	0.013
Modified radical mastectomy	113 (47.5)	119.13 ± 19.82		
Modified radical mastectomy with reconstruction	14 (5.9)	130.36 ± 23.38		
No surgery performed	32 (13.4)	120.75 ± 18.32		
Duration of illness (years)				
<1	106 (44.5)	129.08 ± 17.71	27.373	<0.001
1 ~ 3	62 (26.1)	122.52 ± 17.14		
>3	70 (29.4)	109.83 ± 15.45		
Lymph node or distant metastasis				
No	150 (63.0)	130 ± 18.01	8.706	<0.001
Yes	88 (37.0)	110.3 ± 14.68		
Comorbid chronic diseases				
No	208 (87.4)	122.93 ± 19.49	0.448	0.655
Yes	30 (12.6)	121.23 ± 18.44		

### 3.2 Correlation analysis

A positive correlation was found between family intimacy and adaptability and health-promoting lifestyle (*r* = 0.562, *p* < 0.01) among breast cancer survivors; self-advocacy was also positively correlated with health-promoting behaviors (*r* = 0.575, *p* < 0.01). Additionally, a positive correlation was found between family intimacy and adaptability and self-advocacy (*r* = 0.479, *p* < 0.01). The correlation coefficients among variables are presented in Table 2.

**Table 2:** Pearson’s correlations between study variables.

Variable	Mean (SD)	1	2	3	4	5	6	7	8	9	10	11	12	13	14
1 Interpersonal relationships	21.47 ± 4.52	1													
2 Nutrition	21.86 ± 3.65	0.487**	1												
3 Health responsibility	19.79 ± 4.65	0.5**	0.448**	1											
4 Physical activity	16.51 ± 4.24	0.386**	0.335**	0.373**	1										
5 Stress management	19.54 ± 3.76	0.493**	0.471**	0.49**	0.479**	1									
6 Spiritual growth	23.54 ± 5.05	0.549**	0.446**	0.454**	0.391**	0.698**	1								
7 HPLPII score total	122.7 ± 19.29	0.768**	0.695**	0.734**	0.653**	0.795**	0.81**	1							
8 Family Closeness	86.83 ± 10.18	0.418**	0.281**	0.345**	0.357**	0.387**	0.538**	0.533**	1						
9 Family Adaptability	57.65 ± 8.96	0.459**	0.337**	0.362**	0.375**	0.411**	0.566**	0.573**	0.924**	1					
10 FACESII-CV score total	144.48 ± 18.78	0.446**	0.313**	0.36**	0.373**	0.406**	0.562**	0.562**	0.983**	0.978**	1				
11 Informed decision-making	23.15 ± 5.01	0.464**	0.358**	0.278**	0.247**	0.329**	0.495**	0.497**	0.484**	0.495**	0.499**	1			
12 Effective communication	22.64 ± 3.77	0.563**	0.292**	0.275**	0.254**	0.342**	0.405**	0.477**	0.395**	0.397**	0.403**	0.611**	1		
13 Connected strength	24.39 ± 4.86	0.582**	0.395**	0.326**	0.15*	0.331**	0.41**	0.486**	0.341**	0.371**	0.362**	0.559**	0.562**	1	
14 FSACS score total	70.18 ± 11.58	0.629**	0.414**	0.345**	0.253**	0.391**	0.516**	0.575**	0.479**	0.497**	0.497**	0.867**	0.827**	0.845**	1

**P<0.01; *P < 0.05; HPLP II, Health Promotion Lifestyle Profile II; FACES II – CV, Family Adaptability and Cohesion Evaluation Scales; FSACS, Female Self-Advocacy in Cancer Survivorship Scale.

### 3.3 Evaluation of model fit

**Figure 1:**
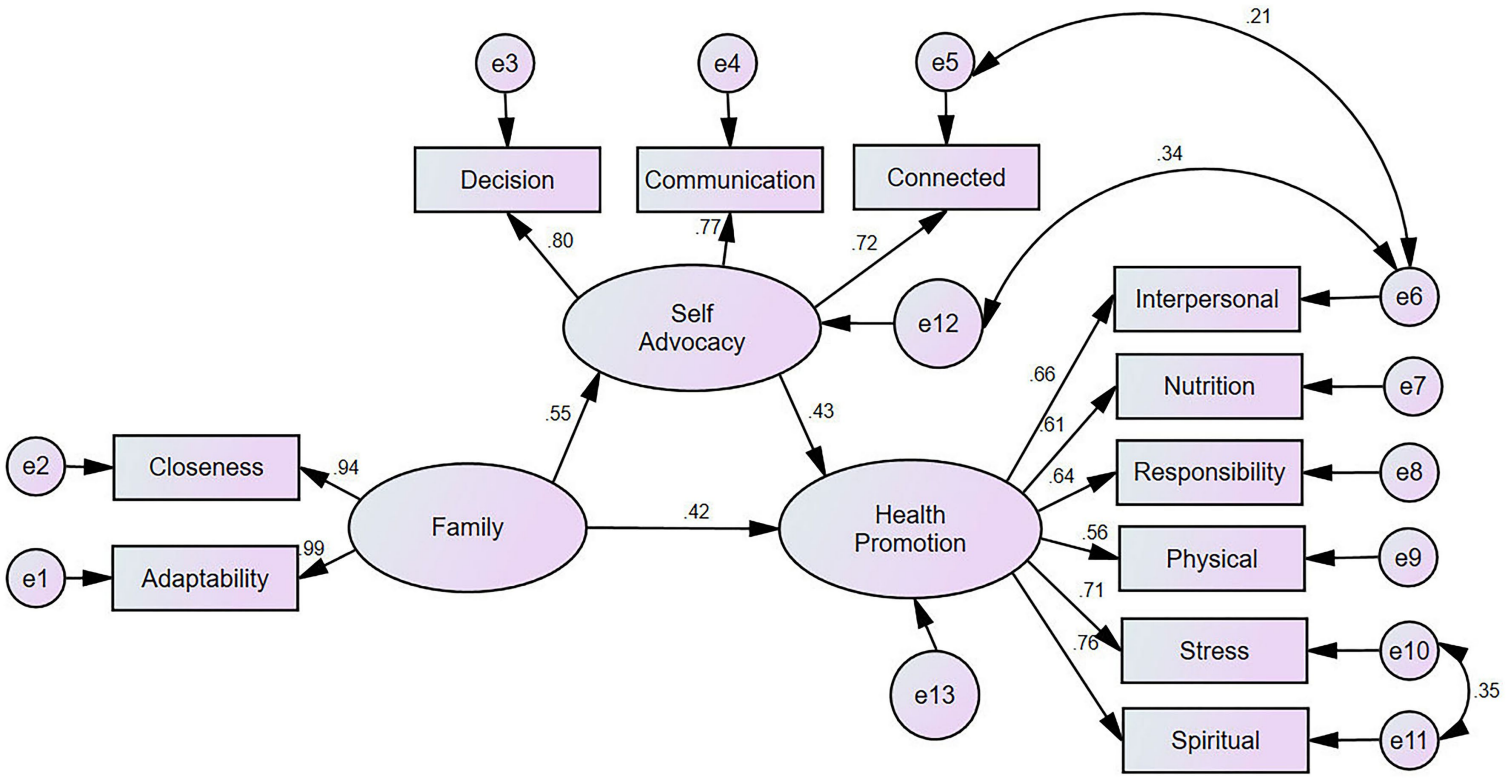
Standardized estimates of the relationships and effect sizes in the structural model. Family: Family Adaptability and Cohesion; Decision, Informed decision-making; Communication, Effective communication; Connected, Connected strength.

A structural equation model was constructed using Amos 24.0 (Figure 1) to examine the relationships between family intimacy and adaptability (independent variables), health promotion (dependent variable), and self-advocacy (mediating variable). Model parameters were estimated using the maximum likelihood (ML) method in AMOS. The following fit indices were employed to assess model fit: the ratio of chi-square to degrees of freedom (X^2^/df), goodness-of-fit index (GFI), root mean square error of approximation (RMSEA), normed fit index (NFI) = 0.946, incremental fit index (IFI), and comparative fit index (CFI) (21). Based on these indices, the model demonstrated a good fit to the data (Table 3).

**Table 3:** Fit indices and evaluation criteria.

Fit indices	X^2^/df	GFI	RMSEA	NFI	IFI	CFI
Evaluation criteria	<3	>0.90	<0.08	>0.90	>0.90	>0.90
Index value	2.148	0.94	0.07	0.946	0.971	0.97

Chi-square/degree of freedom, X^2^/df; goodness-of-fit index, GFI; root mean square error of approximation, RMSEA; normed fit index, NFI; incremental fit index, IFI; comparative fit index, CFI.

### 3.4 Results of model testing

The mediating effect was tested using the Bootstrap method with 2000 resamples and a 95% confidence interval. A significant mediating effect was indicated if the 95% confidence interval for the indirect effect did not include zero; otherwise, no mediating effect was inferred. After controlling for the mediating variable, a direct effect 95% confidence interval excluding zero suggested a partial mediating role, while an interval including zero indicated complete mediation (22). The Bootstrap results revealed a significant indirect effect of self-advocacy between family intimacy/adaptability and health-promoting lifestyle (95% CI [0.112, 0.39]), accounting for 36.4% of the total effect. The direct effect (95% CI [0.226, 0.589]) indicated that self-advocacy partially mediated the relationship (Table 4).

**Table 4:** Mediation effect analysis.

Variable	β	SE	*P*	95%CI	Effect proportion (%)
Total effect	0.653	0.061	0.001	0.527–0.763	–
Direct effect	0.415	0.093	0.001	0.226–0.589	63.60%
Indirect effect	0.237	0.07	0.001	0.112–0.39	36.40%

## 4 Discussion

### 4.1 Current status and factors of health-promoting

The present study revealed a mean health promotion score of 122.7 ± 19.29 among breast cancer survivors, indicating a moderate level of health-promoting behaviors. This finding aligns with results observed in elderly women with coronary heart disease in Hangzhou (125.86 ± 25.65) (23). In contrast, a study by Farideh et al. (24) reported a higher health promotion score of 154.9 ± 2.2 among Iranian pregnant women, suggesting a good level of health promotion. These findings underscore the need for increased attention to health-promoting behaviors in women following a breast cancer diagnosis.

Univariate analysis demonstrated that several demographic and clinical factors, including living status, education level, per capita monthly household income, pathological stage, current treatment regimen, surgical approach, disease duration, and lymph node metastasis status, significantly influenced health behaviors of breast cancer survivors. Consistent with previous research (25, 26), our findings suggest that social support from family members, particularly co-residents, facilitates lifestyle modification and adherence. This may be attributed to the capacity of co-residents to provide supervision and encouragement regarding health behaviors. Furthermore, shared adoption of health-promoting lifestyles among co-residents may mitigate exposure to unhealthy habits, thereby fostering the maintenance of positive health behaviors. The present study also revealed that disease duration impacts health-promoting behaviors in breast cancer survivors. Previous research has indicated that lifestyle modification programs often experience regression to pre-treatment status after initial success, with 30–60% of individuals struggling to sustain behavioral changes (27). However, extending intervention duration, providing problem-solving skills training, and enhancing social support have been identified as effective strategies for improving long-term adherence to health-promoting behaviors (28). The study observed that survivors with advanced disease stages or lymph node metastasis exhibited reduced capacity for adopting health-promoting lifestyles. This may be associated with the recurrent health issues that potentially induce fatigue, anxiety, and depression (5), consequently undermining patients’ confidence in implementing health-promoting behaviors. For these survivors, multidisciplinary team interventions could be implemented to alleviate symptom burden and promptly address negative emotional states.

### 4.2 Correlation analysis

The study results indicated a positive correlation between family intimacy and adaptability with health-promoting behaviors (r = 0.562, *p* < 0.01), aligning with the results of Zhou et al. (29). Zhou et al. demonstrated that stronger family intimacy and adaptability correlated with a greater tendency among cancer survivors to adopt approach-oriented coping strategies. These strategies often involve actively seeking support from family, friends, and healthcare professionals, fostering a sense of control and confidence in their disease management, and promoting proactive engagement in maintaining individual health (29). When illness occurs, highly adaptable families can rapidly adjust to changes, redistribute responsibilities, and alleviate stress on the survivor, enabling them to focus on recovery and health behaviors. Consequently, effective health promotion management for breast cancer survivors should extend beyond the individual to include the family as a crucial target for intervention.

Our study revealed a positive association between self-advocacy and health-promoting behaviors among survivors (*r* = 0.575, *p* < 0.01). Self-advocacy appears to enhance cancer survivors’ sense of responsibility for their health, facilitating their active participation in medical care (30). This may be because individuals who prioritize their well-being are more likely to actively address issues negatively impacting their quality of life, potentially triggering further health-promoting behaviors (31). These findings suggest that healthcare providers should emphasize health education to enhance patients’ knowledge of health management, thereby empowering them to advocate for themselves. Furthermore, granting cancer survivors greater autonomy in health promotion and supporting their initiative in health management may be beneficial.

The results also demonstrated that family intimacy and adaptability positively influenced self-advocacy among breast cancer survivors (r = 0.479, *p* < 0.01). Close family relationships can provide emotional support and security, while family members’ support for autonomous choices and confidence in survivors’ decisions during treatment can foster a positive environment for self-advocacy. Qualitative interviews by Molina et al. (32) with breast cancer survivors indicated that social support from family and friends may facilitate self-advocacy in women, potentially because strong interpersonal relationships provide survivors with the knowledge needed for decision-making and a sense of support. This highlights the importance of healthcare professionals guiding family members to communicate effectively with patients, understand their needs and emotions, and provide help, care, and support, thereby enhancing family intimacy and promoting self-advocacy.

### 4.3 Mediation effect analysis

Female cancer patients are more susceptible to negative emotions and have greater care needs compared to their male counterparts. However, they tend to prioritize compromise during communication (33). This tendency hinders patients from taking initiative in managing their illness and actively engaging in health-promoting behaviors. Self-determination theory posits that the extent to which extrinsic motivation becomes self-determined depends on its level of internalization (14). This suggests that transforming external resources into intrinsic motivation can yield more effective outcomes. As a social resource, family support can only exert its full potential when effectively utilized by cancer survivors. Our study found that self-advocacy mediates the relationship between family cohesion and adaptability and health promotion. Self-advocacy is the process of internalizing and activating resources into actions to overcome cancer- and treatment-related barriers (9). It encourages individuals to actively participate in their health management. Survivors with self-advocacy skills can transform family support into resources for maintaining their health, enabling them to adhere to their treatment preferences and priorities and fostering intrinsic motivation for health promotion.

### 4.4 Limitations

1. The data in this study were primarily collected through scales, which are subject to strong subjective influences. Future research could incorporate objective indicators to further explore the factors influencing health-promoting behaviors in breast cancer survivors.
2. As a quantitative study, this research cannot fully capture the factors influencing health-promoting behaviors in breast cancer survivors. Future studies could combine qualitative interviews to gain deeper insights into the intrinsic motivations and barriers to behavioral changes.
3. This study is cross-sectional and cannot reflect the dynamic process of changes in health-promoting behaviors among breast cancer survivors. Longitudinal studies could be conducted to examine differences in health-promoting behaviors at different time points after diagnosis, thereby identifying vulnerable periods for targeted interventions.

## 5 Conclusion

In summary, family cohesion and adaptability are positively correlated with self-advocacy; self-advocacy is significantly positively correlated with health-promoting behaviors; and family cohesion and adaptability are positively correlated with health-promoting behaviors. Self-advocacy in breast cancer survivors partially mediates the relationship between family cohesion and adaptability and health-promoting behaviors. In clinical practice, healthcare professionals should comprehensively assess survivors’ self-advocacy abilities, family cohesion, and adaptability levels before designing health promotion intervention plans. Additionally, the potential pathways between these variables and health-promoting behaviors should be considered, and multimodal intervention strategies should be developed.

## Data Availability

The raw data supporting the conclusions of this article will be made available by the authors, without undue reservation.
